# C-terminal truncated HBx initiates hepatocarcinogenesis by downregulating TXNIP and reprogramming glucose metabolism

**DOI:** 10.1038/s41388-020-01593-5

**Published:** 2020-12-15

**Authors:** Yu Zhang, Qian Yan, Lanqi Gong, Hang Xu, Beilei Liu, Xiaona Fang, Dandan Yu, Lei Li, Ting Wei, Ying Wang, Ching Ngar Wong, Zhaojie Lyu, Ying Tang, Pak Chung Sham, Xin-Yuan Guan

**Affiliations:** 1 Department of Clinical Oncology, The University of Hong Kong, Hong Kong, Hong Kong; 2 State Key Laboratory for Liver Research, The University of Hong Kong, Hong Kong, Hong Kong; 3 Research Center of Medical Science, Guangdong Provincial People’s Hospital, Guangdong Academy of Medical Sciences, Guangzhou, 510030 China; 4 Department of Clinical Oncology, The University of Hong Kong-Shenzhen Hospital, Shenzhen, 518053 China; 5 Department of Psychiatry, Li Ka Shing Faculty of Medicine, The University of Hong Kong, Hong Kong, Hong Kong; 6 Department of Biology, The Southern University of Science and Technology, Shenzhen, 518055 China; 7 Department of Oncology, Zhujiang Hospital, Southern Medical University, Guangzhou, 510282 China; 8 State Key Laboratory of Oncology in Southern China, Sun Yat-sen University Cancer Center, Guangzhou, 510060 China

**Keywords:** Cancer genomics, Liver cancer

## Abstract

Chronic hepatitis B virus (HBV) infection is strongly associated with the initiation and development of hepatocellular carcinoma (HCC). However, the genetic alterations and pathogenesis mechanisms remain significantly unexplored, especially for HBV-induced metabolic reprogramming. Analysis of integration breakpoints in HBV-positive HCC samples revealed the preferential clustering pattern within the 3′-end of X gene in the HBV genome, leading to the production of C-terminal truncated X protein (Ct-HBx). In this study, we not only characterized the oncogenic role of two Ct-HBx (HBx-120 and HBx-134) via in vitro and in vivo functional assays but also deciphered their underlying molecular mechanisms. Gene expression profiling by transcriptome sequencing identified potential targets of Ct-HBx and novel malignant hallmarks such as glycolysis, cell cycle, and m-TORC1 signaling in Ct-HBx-expressing cells. TXNIP, a well-established regulator of glucose metabolism, was shown to be downregulated by Ct-HBx and play a pivotal role in Ct-HBx-mediated HCC progression. Suppression of TXNIP is frequently observed in HCC patients with Ct-HBx expression and significantly (*P* = 0.015) correlated to a poorer prognosis. Re-introduction of TXNIP attenuated the metabolic reprogramming induced by the Ct-HBx and inhibited the tumor growth in the mice model. Further study suggested that Ct-HBx could downregulate TXNIP via a transcriptional repressor nuclear factor of activated T cells 2 (NFACT2). Collectively, our findings indicate that TXNIP plays a critical role in Ct-HBx-mediated hepatocarcinogenesis, serving as a novel therapeutic strategy in HCC treatment.

## Introduction

Chronic hepatitis B virus (HBV) infection has been well established as an independent risk factor in the occurrence and progression of hepatocellular carcinoma (HCC) [1] by epidemiological studies, accounting for 60% cases worldwide [2], which is even higher in East Asian regions where HBV infection is endemic [3]. The emerging advances suggest that HCC progression involves multiple HBV-induced alterations including liver fibrosis/cirrhosis [4, 5], genomic integration of viral DNA [6, 7], and the oncogenic potential of HBV-encoding proteins, such as X protein (HBx) [8].

We and others have previously reported that the rearrangement of the HBV sequence was detected in almost all HCC samples upon integration into the host genome [9, 10]. In addition, the oncogenic effects of the subsequent C-terminal truncated HBx protein (Ct-HBx) have been systematically reviewed [8, 11–13]. However, the molecular mechanism of HBV-induced HCC initiation remains largely unexplored. Hence, we investigated and confirmed the breakpoints at the 3′-end X gene is a tumor-specific instead of a structure-dependent event in the exploration cohort from the public database and validation cohort of our own clinical samples. We later conducted RNA sequencing to compare gene expression profiles among immortalized liver cells transfected with different HBx mutants. Based on the transcriptomics screening and profiling, thioredoxin interacting protein (TXNIP) has been identified and characterized as the downstream target of Ct-HBx, acting as a negative regulator of aerobic glycolysis during HCC progression.

Metabolic reprogramming is one of the significant malignant hallmarks in cancer progression, satisfying the energy demand of cancer cells for their exponential proliferation capability via increasing glucose uptake [14]. There are substantial amount of virus-mediated metabolism reprogramming mechanisms identified in varying types of cancers, including herpesvirus-related Kaposi’s sarcoma [15, 16], Epstein–Barr virus (EBV)-related nasopharyngeal carcinoma [17, 18], Adenovirus E4ORF1-related lung cancer [19], and HPV-related cervical cancer [20].

TXNIP is a major regulator of the cellular reduction-oxidation system, it also acts as a glucose sensor and negative regulator of glucose uptake in response to dynamic changes in the microenvironment [21–23]. The tumor-suppressive role of TXNIP via modulating metabolic reprogramming has been studied in pancreatic cancer [24] and breast cancer [25]. However, the role of TXNIP in hepatocarcinogenesis is quite controversial. Some papers claimed that full-length HBx may promote HCC invasion via upregulating TXNIP [26], while other papers showed that Txnip-deficient mice had increased incidence of HCC [27]. Although HCC is characterized as one of the most hypoxic solid tumors with the average oxygen content of 0.8%, little is known about the metabolic-regulating function of TXNIP in HBV-induced HCC. In the present study, integrated bioinformatics analysis of whole-genome sequence and both in vivo and in vitro functional assay confirmed the oncogenic function of Ct-HBx in HBV-induced HCC. We identified TXNIP as the downstream target regulating aerobic glycolysis based on transcriptomics screening and profiling. Re-introduction of TXNIP in Ct-HBx-expressing liver cells successfully eliminated the aberrant glycolytic flux indicated by aerobic glycolysis assay. Downregulation of TXNIP has clinical significance both in the TCGA cohort and our own cohort. We also demonstrated that Ct-HBx transactivates NFATC2, thus facilitating the transcriptional repression of TXNIP.

## Results

### HBV breakpoints within the HBV genome are clustered at the 3′-end of X gene in HCC cells

To better characterize the relationship between HBV integration and HCC development, most previous papers focused on the recurrent and preferential insertion sites on the host genome. Here we are curious about the HBV breakpoints within the virus genome. HBV integration sites can be identified by paired-end reads that are mapped on both HBV and the human genome. In order to examine the precise distribution of HBV integration breakpoints, we analyzed the whole-genome sequencing (WGS) data for 29 HCC samples from ERP001196 in the European Genome-phenome Archive (EGA) [6]. WGS data were first mapped on the HBV genome (NC_003977) using BLAT [28], and the mapping reads with more than 20 bp were further mapped on the human genome (hg19). Chimeric reads that have at least one end mapped on both HBV and human genome, as well as spanning reads that have only one end mapped on the HBV genome and the other end on the human genome, were selected for downstream characterization. The integration site with at least one chimeric read and spanning read has a higher confidence level for being identified as the position of HBV breakpoint. However, in this study, we maintained the data of all identified integration sites to enhance sensitivity, and all supporting reads were considered for further analysis.

**Fig. 1:**
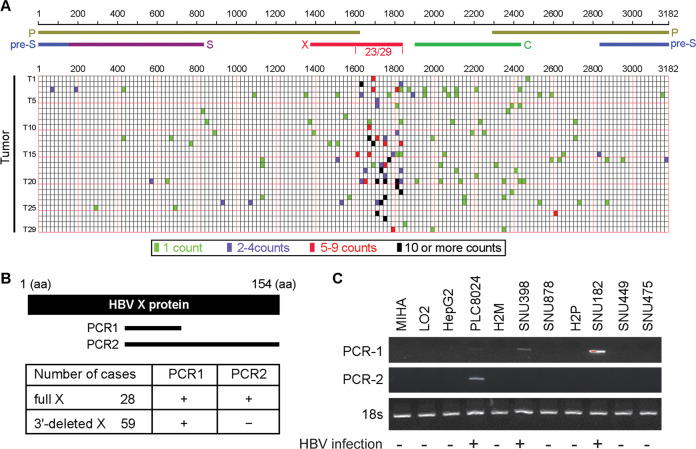
HBV breakpoints within the HBV genome were clustered at 3′-end X gene region in HCC samples. **A** The frequency of integration breakpoints within the HBV genome (NC_003977) is indicated by bars in 29 cases of tumor samples. Integration sites with different supporting counts are indicated by different colors. Genes encoding HBV pre-surface (blue), surface (purple), polymerase (yellow), core (green), and X (red) protein are also shown. **B** Two pairs of primers are designed for the detection of full-length and 3′-end deleted X gene. 66F + 100R flanks 360 nucleotides (PCR1), 66F + 154R flanks 462 nucleotides (PCR2). Expression of full-length and 3′-end deleted X gene in 87 HBV-related HCC samples were detected by qRT-PCR. **C** Expression pattern of both full-length and 3′-end deleted X gene in a panel of hepatoma cell lines was detected by RT-PCR, 18 s served as the internal reference.

Distribution pattern of HBV breakpoints on virus genome revealed that HBV breakpoints in HCC are frequently clustered around 1600–1837 bp region (Fig. 1A), where the 3′-end of X gene is located (240/464, *p* = 5.2 × 10^8^ by Wilcoxon rank-sum test), especially for those with more than 5 supporting reads. Diverse models strongly support that HBV integration is an early event and remain relatively stable in hepatocarcinogenesis, our data strongly supported the well-recognized monoclonal HCC model. So, we hypothesized that such preferred distribution of breakpoints may contribute to HCC initiation and progression in virtue of the generation of Ct-HBx protein in liver cells.

### Ct-HBx is higher expressed in HBV-related HCCs compared to full-length HBx

The expression of full-length HBx and Ct-HBx was compared in 87 HCC samples from patients who had serological positive HBsAg. We constructed 66F/100R primers to amplify the clones (PCR1) expressing both Ct-HBx and full-length HBx, and 66F/154R primers to detect clones (PCR2) only expressing full-length HBx (Fig. 1B). In all the 87 HBV-positive HCC samples, full-length HBx was detected in 28/87 cases (32.2%), whereas the Ct-HBx was detected in the rest 59 cases (67.8%) (Fig. 1B). The expression of full-length (462 bp) and 3′-end deleted (360 bp) HBV X genes were tested in 9 HCC cell lines and two immortalized normal liver cell lines by RT-PCR, and 3′-end deleted and full-length X genes were detected in 2/3 (SNU398 and SNU182) and 1/3 (PLC-8024) of HBV-positive cell lines (Fig. 1C). The results were very consistent with previous reports that human HCC cell line PLC/PRF/5 (PLC8024), SNU182, and SNU398 each containing one or multiple copies of HBV genome fragments.

### Ct-HBx promotes cell proliferation in liver cells

**Fig. 2:**
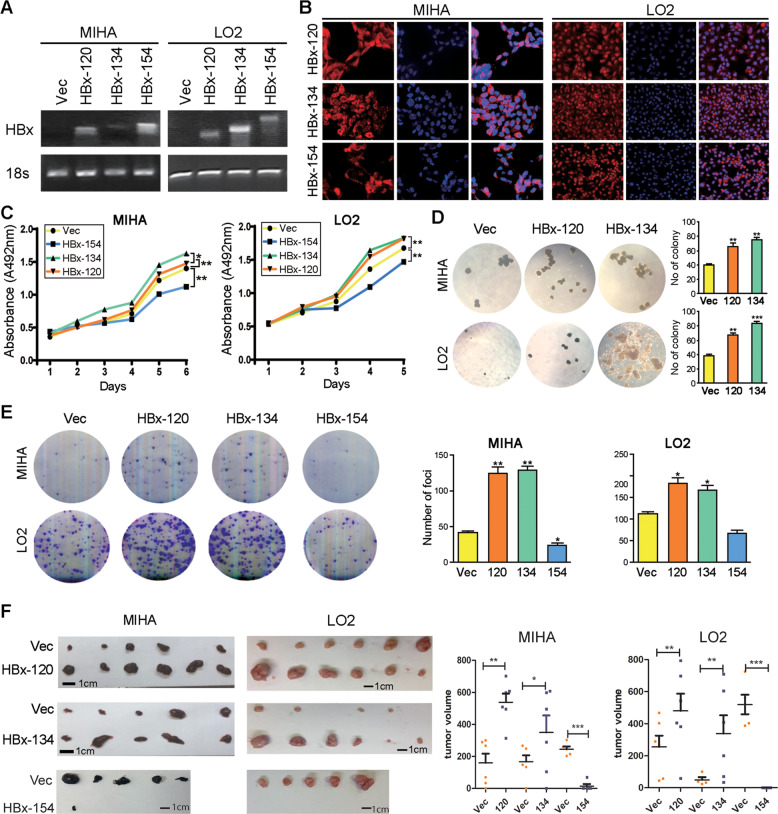
Ct-HBx promotes hepatocarcinogenesis. **A** RT-PCR was applied for validation of overexpression of HBx at the genomic level, 18 s served as an internal reference. **B** Immunofluorescence staining was applied for validation of overexpression of HBx at the protein level and the subcellular location of HBx was also indicated. **C** Ct-HBx promotes cell viability as indicated by XTT value in both LO2 and MIHA cells compared to the control group, full-length HBx showed the opposite effect. **D** More and bigger colony formation were shown in Ct-HBx-expressing cells compared with those in vector cells, as indicated in soft agar assay. No colony formed in full-length HBx-expressing cells. **E** Foci formation frequency was significantly increased in Ct-HBx-expressing MIHA and LO2 cells. Both representative pictures and calculated numbers were shown. **F** In vivo xenograft model showed bigger tumor formation in mice subcutaneously injected with Ct-HBx-expressing MIHA and LO2 cells compared with the control group.

Our previous data demonstrated that two 3′-end deleted X genes encoding Ct-HBx (HBx-120 and HBx-134) were most frequently detected in HBV-positive HCC samples [9, 11]. To characterize the oncogenic effect of varied HBx fragments, full-length X gene (encoding HBx-154) and two 3′-end deleted X genes (encoding HBx-120 and HBx-134) were cloned and stably transduced into two HBV-negative immortalized normal liver cell lines LO2 and MIHA. RT-PCR (Fig. 2A) and immunofluorescence staining (Fig. 2B) were conducted to validate the ectopic overexpression of HBx at both RNA and protein levels, and HBx was confirmed localized to the cytoplasm as well as the nucleus (Fig. 2B). Compared to the control group (empty vector-infected cells), expression of HBx-134 and HBx-120 could significantly enhance cell viability, whereas HBx-154 exhibited an opposite effect (Fig. 2C). We further conducted foci formation and soft agar assays to test the effects of HBx variants on anchorage-independent and anchorage-dependent cell proliferation capability, and the results showed that both anchorage-dependent and independent cell proliferation were dramatically enhanced in CT-HBx-expressing cells compared with control and full-length HBx groups (Fig. 2D and E). Interestingly, truncated HBx-infected cells also possessed elevated metastatic and invasive abilities, demonstrated by cell migration and invasion assays (Supplementary Fig. 1).

In the in vivo mice model to explore tumorigenic potential of Ct-HBx, tumor volumes from mice injected with the truncated HBx-expressing cells were predominantly larger than those injected with vector-infected cells (Fig. 2F). Tumor formation only showed in one mouse incubated with full-length HBx-expressing cells. The in vivo xenograft formation assay was repeated in LO2 cells and the result was consistent (Fig. 2F).

### RNA sequencing identifies metabolism reprogramming regulators in Ct-HBx-expressing LO2 cells

**Fig. 3:**
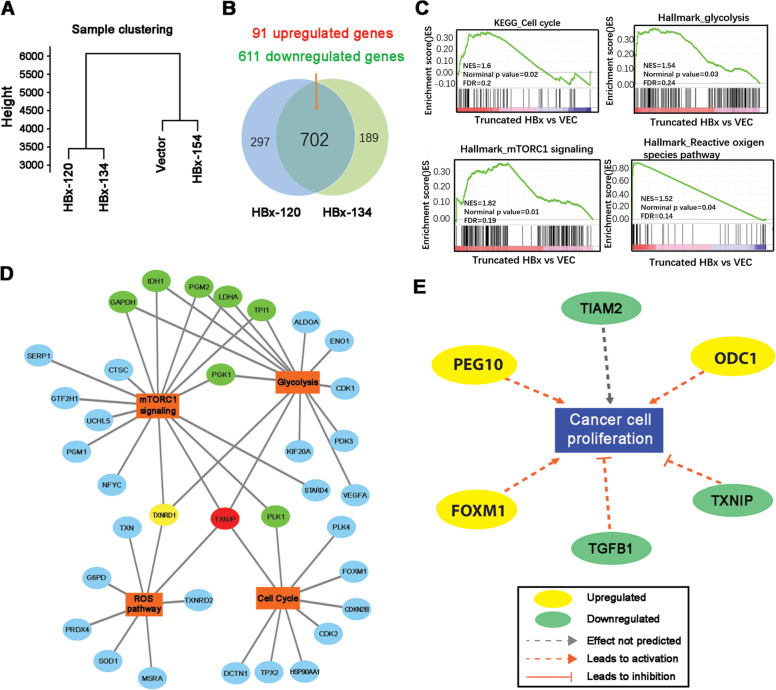
Transcriptome sequencing identified TXNIP as the downstream target regulating glucose metabolism. **A** K-means clustering analysis on all samples using total genes revealed gene expression profile was quite similar between VEC and HBx-154 expressing LO2 cells. **B** Venn diagram showing the number of differentially expressed genes separately between VEC vs HBx-120 and VEC vs HBx-134 in LO2 cells based on the following cut-off line: fold change > 1.5, FDR (adjust *P*-value) < 0.05. The overlapping area showing the commonly regulated genes. **C** Gene set enrichment analysis (GSEA) demonstrated 4 hallmarks significantly enriched in Ct-HBx-expressing LO2 cells. **D** A selection of the most significantly regulated genes in each GSEA sets above was displayed by Cytoscape. Genes involved in two networks are labeled with green, genes involved in three out of four networks are labeled with yellow. Gene involved in all four networks is labeled with red. **E** Network enrichment analysis of the deregulated genes in profile 3 by Ingenuity Pathway Analysis (IPA) revealed TXNIP plays a causal role in the proliferation of liver cells.

To explore the molecular mechanism of Ct-HBx-mediated hepatocarcinogenesis, we conducted transcriptome sequencing to compare gene expression profiles among the 4 LO2 variants, including HBx-154, HBx-134, HBx-120, and empty vector-transduced cells. After quality control and alignment to the hg38 reference genome, the expression level of each gene was normalized as fragments per kilobase of transcript per million mapped read (FPKM). Based on the selection criteria of fold change > 1.5 and FDR < 0.05, the number of genes considered differentially expressed among the four LO2 variants was shown in Supplementary Fig. 2a. Hierarchy clustering analysis of the four LO2 variants revealed that the gene expression profiles for HBx-120 and HBx-134-expressing cells were closely related, thus being grouped into one cluster (Fig. 3A), which supported the results from the functional assays. Tendency analysis using Short Time-series Expression Miner software also demonstrated that 596 genes enriched in profile 3 (Supplementary Fig. 2b) were significantly and consistently downregulated in HBx-134 and HBx-120 expressing cells, but remained unchanged in full-length HBx group, compared to vector-transduced cells. We identified 91 commonly upregulated genes and 602 downregulated genes between two Ct-HBx and vector-transduced cells (Fig. 3B). Gene set enrichment analysis (GSEA) showed that cell cycle, glycolysis, m-TORC1 signaling, and reactive oxygen species pathway are the top enriched hallmarks in truncated HBx-expressing cells (Fig. 3C). To decipher the molecular drivers in those processes, we constructed an interaction network based on the most significant genes in each gene set using Cytoscape (Fig. 3D). Since the interplay between m-TORC1 and aerobic glycolysis has been well-addressed in many types of cancers [29, 30], we found some co-regulators in these two networks. One of the vital regulators, the thioredoxin interacting protein (TXNIP), was enriched in 4 regulatory networks mediated by Ct-HBx and has been recognized as a well-established regulator of glucose metabolism. Meanwhile, TXNIP was also found highlighted in the network of “Proliferation of liver cancer” when we performed Ingenuity Pathway Analysis (IPA) using differentially expressed genes in profile 3 (Fig. 3E). Therefore, TXNIP was considered as a potential downstream target of Ct-HBx and regulator of glucose metabolism in HBV-induced HCC.

### TXNIP is transcriptionally downregulated by Ct-HBx and low TXNIP expression is associated with poor clinical outcome in HCC

**Fig. 4:**
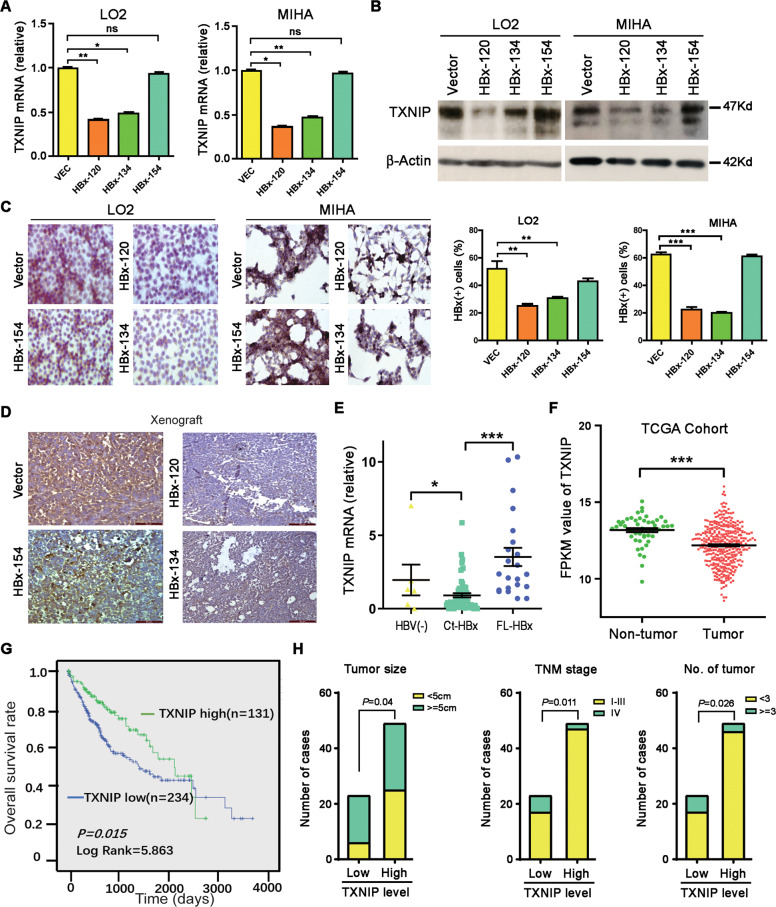
TXNIP is transcriptionally downregulated by Ct-HBx and low TXNIP expression is associated with poor clinical outcomes in HCC. **A** The expression level of TXNIP was downregulated at the genomic level in Ct-HBx group (HBx-120 and HBx-134) compared with vector and full-length group (HBx-154) as shown by qRT-PCR. **B**, **C** The expression of TXNIP is significantly lower at the protein level in Ct-HBx (HBx-120 and HBx-134) transduced cells, compared to full-length (HBx-154) and vector group as indicated by western blotting (**B**) and immunohistochemistry (**C**). **D** Representative IHC images of TXNIP staining in mice xenografts induced by LO2 cells infected with HBx-120,134, 154, and empty vector. **E** Realtime-PCR result showed the mRNA expression of TXNIP in HBV-negative HCC patients (*n* = 5), Ct-HBx-expressing HCC patients (*n* = 55) and full-length HBx (FL-HBx) expressing HCC patients (*n* = 22). **F** The expression of TXNIP is significantly lower in primary HCC samples (*n* = 369) compared with normal liver tissues (*n* = 51) in the TCGA cohort. **G** Kaplan–Meier analysis of the correlation between TXNIP expression and overall survival rate in TCGA patients of HCC. **H** Correlation regression analysis showed expression of TXNIP significantly associated with tumor size (Pearson *χ*^2^ test, *P = 0.04), tumor number (Pearson *χ*^2^ test, *P ≤ 0.026) and TNM stage (Pearson *χ*^2^ test, *P ≤ 0.011).

To confirm whether the expression of TXNIP mRNA is regulated by the truncated HBx, qPCR was performed to compare the TXNIP levels among the various HBV X genes infected cells as well as control cells. The results found that TXNIP was significantly downregulated in HBx-120 and HBx-134 transduced cells, whereas such expression alteration was not observed in HBx-154 transduced cells (Fig. 4A). The expression of TXNIP was then detected at the protein level and the result was consistent (Fig. 4B). Immunohistochemical (IHC) staining was performed on HBx-transduced cells as well as tumor xenografts in nude mice induced by these cells. A lower percentage of TXNIP-positive cells was stained in the truncated HBx-expressing LO2 and MIHA cells, compared to the HBx-154 and vector-expressing cells (Fig. 4C). The expression of TXNIP was hardly detected in tumor xenografts induced by the truncated HBx-expressing cells (Fig. 4D). The relationship between Ct-HBx and TXNIP was further validated in HBV-positive and negative HCC clinical samples. The result found that the expression level of TXNIP was much lower in patients with Ct-HBx expression than either patient with full-length HBx expression or HBV-negative patients (Fig. 4E). Informative data was also obtained from 369 HCC samples and 51 non-tumor samples collected from The Cancer Genome Atlas (TCGA), where the downregulation of TXNIP was observed in HCC samples compared to non-tumor samples (Fig. 4F). In addition, the data from TCGA was exploited for Kaplan–Meier analysis of clinical outcome and the result showed that downregulation of TXNIP was significantly (*P* = 0.015) associated with a poorer prognosis for HCC patients (Fig. 4G). The Clinical pathology study of our clinical cohort found that the expression of TXNIP was significantly associated with tumor size, tumor nodules, and TNM stage (Fig. 4H). Taken together, these findings suggest that TXNIP is a potential tumor-suppressing gene, which can be downregulated by Ct-HBx.

### Re-introduction of *TXNIP* could inhibit the metabolic reprogramming induced by Ct-HBx

**Fig. 5:**
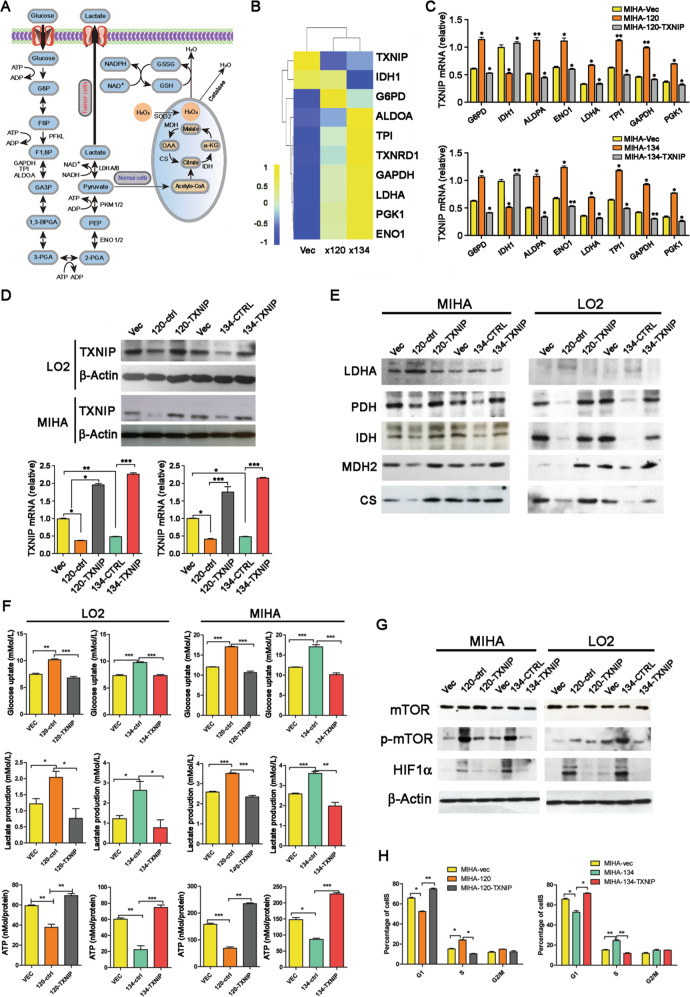
TXNIP induced glucose metabolism reprogramming from glycolysis to mitochondrial respiration. **A** Schematic representation of the biological process of glucose metabolism in normal cells and cancer cells. **B** Heatmap showing the relative expression level of several genes involved in glucose metabolism in Ct-HBx and vectors containing samples as indicated by RNA sequencing, each matrix representing the relative expression level of an individual gene, high and low expression are indicated by yellow and blue color. **C** The expression level of the gene panel indicated above was validated by qRT-PCR in MIHA cells transduced with truncated HBx mutants compared with vector group, also the expression was further compared after re-introduction of TXNIP into Ct-HBx expressing cells. **D** Re-introduction of TXNIP into Ct-HBx (HBx-120, HBx-134) expressing cells was confirmed at the protein and genomic level by western blotting and qRT-PCR. **E** The expression level of several key enzymes and molecules participated in glycolysis and Krebs cycle are determined by western blotting. The expression of internal reference ß-actin can be referred to in Fig. 5d. **F** Level of glucose uptake, lactate secretion, and relative ATP production activity were compared among vector, Ct-HBx as well as TXNIP overexpression samples. **G** The activation of the mTOR-HIF1α axis was detected by western blotting, ß-actin was used as an internal reference. **H** Analysis of cell distribution in each stage of the cell cycle in each transfected MIHA cells.

As GSEA analysis suggests that Ct-HBx may induce glycolysis, we next checked the effect of Ct-HBx on key regulators in glucose metabolism (Fig. 5A) in our RNA-seq data. A gene profile consisting of seven key enzymes and molecules (G6PD, ALDOA, TPI, GAPDH, LDHA, PGK1, and ENO1) involved in glycolytic cascade was shown consistently upregulated in the truncated HBx-expressing LO2 cells, except for IDH1, which participated in oxidative phosphorylation through Kreb’s cycle (Fig. 5B). To validate our gene profile generated by RNA sequencing, the expression of these deregulated genes was quantified in both LO2 and MIHA cells by qPCR, and the result was consistent (Fig. 5C, Supplementary Fig. 3a). Although TXNIP is a well-recognized glucose sensor and regulator in glucose metabolism in many types of cancers, its role in HCC has not been reported. Thus, we re-introduced TXNIP in truncated HBx-expressing cells to study whether the alteration of glucose metabolism induced by Ct-HBx could be rescued by TXNIP overexpression. qRT-PCR and western blotting were utilized to confirm the overexpression of TXNIP at the RNA and protein levels (Fig. 5D). The key molecules indicated above were significantly alleviated after the re-introduction of TXNIP into truncated HBx-expressing cells (Fig. 5C, Supplementary Fig. 3a). The expression changes of these regulators in glycolysis cascade in RNA derived from truncated HBx-induced mice xenograft was also consistently eliminated upon stable re-introduction of TXNIP (Supplementary Fig. 3b).

To investigate whether there was a metabolism reprogramming from mitochondrial respiration to aerobic glycolysis induced by Ct-HBx through downregulation of *TXNIP*, we next detected protein expression of several enzymes involved in the glycolytic cascade. The result demonstrated that the expression of LDHA, a key enzyme converting pyruvate to lactate, increased obviously in truncated HBx-transduced MIHA and LO2 cells. However, four other key enzymes (PDH, MDH2, CS, and IDH1), which drive glucose metabolism to acetyl-CoA generation and then subjected to the tricarboxylic TCA cycle in mitochondrial, were dramatically downregulated in the truncated HBx-transfected MIHA and LO2 cells (Fig. 5E). Moreover, these expressional alterations could be alleviated by the re-introduction of TXNIP into truncated HBx-transduced cells in both LO2 and MIHA cells. We then studied the effects of TXNIP on glucose uptake, lactate production, and ATP production in truncated HBx-transduced cells. The results found that glucose uptake and lactate production were significantly increased in truncated HBx-transduced MIHA and LO2 cells, accompanied by decreased ATP production (Fig. 5F), indicating the truncated HBx greatly accelerated the glycolytic activity. As expected, the change of these three major biochemical parameters for glycolytic activity was reversed by the re-introduction of TXNIP (Fig. 5F).

Emerging evidence in recent years has identified mTORC1 signaling as one of the master regulators of aerobic glycolysis in cancer cells. mTORC1 may activate HIF1α and subsequently induces metabolic reprogramming [30–32]. Furthermore, TXNIP has been reported as a suppressor of mTORC1 activity by binding to and stabilizing its negative regulator [33]. Therefore, mTOR activity and expression of HIF1α were also tested in the present study. Western blotting results showed that phosphorylated mTOR (p-mTOR) and its downstream HIF1α were upregulated in the truncated HBx-transduced MIHA and LO2 cells (Fig. 5G), which could be effectively inhibited after the re-introduction of TXNIP. Cell cycle analysis showed that less truncated HBx-transduced LO2 and MIHA cells were arrested at G1-S checkpoint, as there was a significant increase in the distribution of cells at S stage (Fig. 5H, Supplementary Fig. 3c) and decreased number of cells were detected at G1 stage. Re-introduction of TXNIP impaired the accelerated cell cycle progress induced by Ct-HBx. These findings suggested that Ct-HBx may serve as a supplementary route promoting glucose metabolism in HCC cells through the TXNIP-mTORC1-HIF1α axis.

### Ct-HBx induced transactivation of TXNIP through NFATC2

**Fig. 6:**
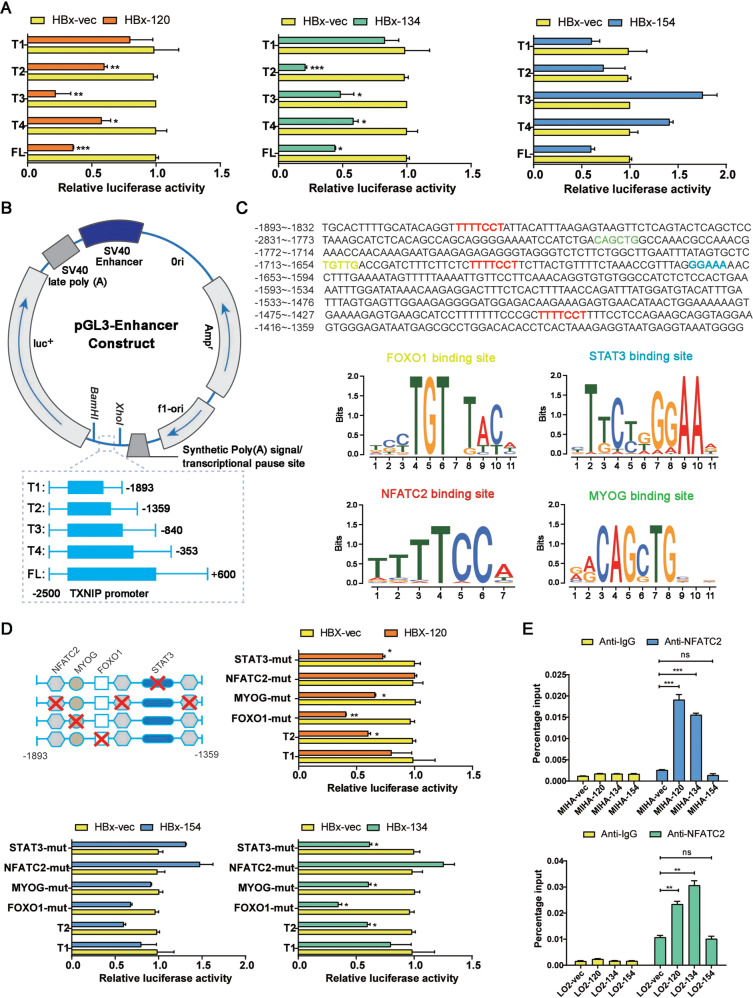
Truncated HBx downregulates the expression of TXNIP by transactivation through NFATC2. **A** Relative luciferase activity in each of the truncated mutants of TXNIP promoter (T1, T2, T3, and T4), the full length of TXNIP promoter (FL), and HBx containing samples indicated by dual-luciferase reporter assay. **B** Schematic illustrator of the luciferase reporter constructs containing different lengths of truncated mutants of TXNIP promoter. **C** Prediction of the *cis*-regulatory elements between nt-1893 and nt-1359 on the promoter of TXNIP by JASPAR revealed three NFATC2 binding sites, one MYOG binding site, one STAT3 binding site, and one FOXO1 binding site. **D** Relative luciferase activity after mutagenesis of the candidate binding sites indicated by dual-luciferase reporter assay, TI and T2 are served as a negative and positive control. Mutagenesis of MYOG, STAT3, and FOXO1 showed little change in the luciferase activity, and NFATC2 is essential for the transcription downregulation induced by truncated HBx. **E** The binding of NFATC2 to the promoter of TXNIP mediated by truncated HBx was validated by the CHIP assay.

Since HBx may serve as a transactivator that activates or repress a variety of viral and cellular promoters and enhancers [34], we next investigated whether the Ct-HBx regulates the expression of TXNIP by transactivation. Luciferase reporter assay showed that the luciferase activity of PGL3-TXNIP was significantly repressed in 293FT cells co-transduced with two Ct-HBx variants (HBx-120 and HBx-134), but not with full-length HBx (HBx-154) and empty vector (Fig. 6A). Transactivation needs *cis*-regulatory for binding activity, to identify the precise binding site on the TXNIP promoter in response to the truncated HBx transactivation. A series of 3′ deletion of TXNIP promoter was generated and cloned into PLG3-enhancer plasmid (Fig. 6B). Luciferase reporter assay was then repeated in 293FT cells co-transduced with different deletion mutants of TXNIP promoter and HBx containing plasmids, results showed that deletion from full length to T2 (+660/−1359) had no effect on the TXNIP promoter activity induced by the truncated HBx, further deletion from T2 to T1 (−1359/−1893) significantly impeded the repressive activity of TXNIP promoter, indicating that regions from T1 to T2 on TXNIP promoter is crucial for the transactivation of TXNIP mediated by the truncated HBx (Fig. 6A). Prediction analysis of the *cis*-regulatory elements between −1893 and −1359 region on TXNIP promoter using TRRUST and Genomatix revealed one STAT3 binding site, one FOXO1 binding site, one MYOG binding site, and 3 NFATC2 binding sites (Fig. 6C). Upon applying the site-directed mutagenesis to each binding site, luciferase reporter assay showed that inhibition of the suppressive TXNIP promoter activity in response to Ct-HBx was observed in NFATC2-mutated cells. Mutagenesis of the other three binding sites showed no effect (Fig. 6D). The NFATC2 binding activity was further validated using a Chip assay (Fig. 6E). The results clearly demonstrated that binding of NFACT2 to the promoter of TXNIP is required for the suppression of TXNIP expression mediated by Ct-HBx. Next, we checked and found that the mRNA level of NFATC2 showed no significant differences among Ct-HBx, full-length or empty vector containing LO2 cells as indicated by RNA sequencing, however, previous studies did provide some evidence showing HBx may activate NFATC2 involving nuclear translocation of NFATC2 from the cytoplasm [35].

### Re-introduction of *TXNIP* induced cell growth arrest both in vitro and in vivo

**Fig. 7:**
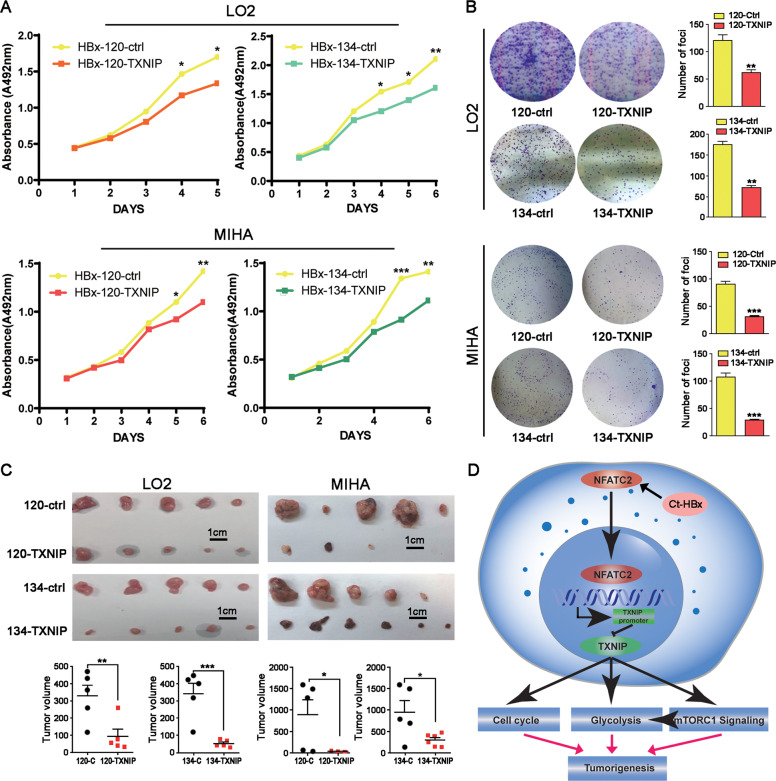
Re-introduction of TXNIP significantly caused tumor growth arrest in vitro and in vivo. **A** The cell proliferation ability of Ct-HBx was eliminated by the introduction of TXNIP in both MIHA and LO2 cells as indicated by XTT assay. **B** Overexpression of TXNIP abrogated the increased colony formation rate and size induced by Ct-HBx in both MIHA and LO2 cells. **C** Re-introduction of TXNIP induced the tumor growth arrest after subcutaneous injection of both LO2 and MIHA cells. **D** Schematic diagram demonstrating the oncogenic role and mechanism of Ct-HBx and TXNIP in HBV-related HCC. Basically, Ct-HBx downregulates the expression of TXNIP by transactivation through NFATC2. Downregulated TXNIP promotes hepatocarcinogenesis by inducing metabolic reprogramming to aerobic glycolysis in a mTORC1-dependent and independent manner.

Since the re-introduction of TXNIP successfully rescued the glucose metabolism promoting the effect of Ct-HBx, we further characterized whether it could arrest tumor cells growth. In vitro results from XTT and foci formation assay showed that the re-introduction of TXNIP significantly reduced cell proliferation (Fig. 7A and B) in both LO2 cells and MIHA cells. In vivo mice xenograft growth assay exhibited that re-introduction of TXNIP successfully inhibited tumor growth as compared with the control group (Fig. 7C). In summary, our data suggested that Ct-HBx downregulated the expression of TXNIP by transactivation through the transcription repressor NFATC2, then TXNIP exerted its tumor-suppressing function in HBV-induced HCC by negative regulating glycolytic metabolism (Fig. 7D).

## Discussion

During the past decades, HBV integration has been deemed as one of the major reasons that facilitate the initiation and development of HCC. Single-cell genome sequencing has confirmed that in monoclonal HCC models, HBV integration pattern remains extremely unchanged during hepatocarcinogenesis. In the present study, bioinformatics analysis of HCC samples from WGS revealed that HBV integration breakpoints preferentially clustered within the 3′-end X gene. We then cloned the two 3′-end deleted X mutants (coding Ct-HBx: HBx-134 and HBx-120) which are most frequently present in clinical samples and explored the oncogenic mechanism of the Ct-HBx via transcriptome sequencing and profiling. The gene expression profiles recognized aerobic glycolysis as one of the most significantly altered biological processes and *TXNIP* as a key downstream driver in truncated HBx-expressing cells.

Emerging advances have suggested that metabolic reprogramming in cells primarily contributes to carcinogenesis. Therefore, several diagnostic strategies have been developed and optimized based on the “Warburg effect.” The maximum standardized uptake value (SUV_max_) of malignant cells indicated by PET-CT scanning is strongly associated with tumor burden, pathological features and prognosis in many cancer types [36, 37]. The metabolic alterations from oxidative phosphorylation to aerobic glycolysis meets the increased biosynthetic demand by rapid proliferating cells and provides a favorable microenvironment for tumor metastasis [14, 38, 39]. In HCC, several critical molecules involved in the multi-step process of glucose metabolism have been characterized as biomarkers for prognosis prediction and therapeutic selection, such as LDH [40], PKM2 [41], and PGK1 [42]. Meanwhile, driving pathways or vital regulators in HCC metabolism have been addressed, including the AMPK signaling pathway [43], PI3K/Akt/mTOR pathway [44], and Myc-HIF1α axis [45]. Virus-induced carcinogenesis via metabolic reprogramming in many types of cancers has been supported by emerging advance [24, 46, 47], but few studies have focused on the metabolic alternations in HBV-induced HCC.

TXNIP is a well-recognized regulator of the redox process and a glucose sensor. TXNIP has been reported critical in diabetes mellitus (DM) by influencing glucose metabolism in pancreatic ß cells. Recently, it has been characterized as a tumor suppressor gene that can negatively regulate aerobic glycolysis in hematologic malignancies and solid tumors [25, 26, 48]. Lower expression of TXNIP has also been associated with the activation of mTOR signaling module which plays a decisive regulatory role in facilitating glucose uptake [49]. Based on gene expression profiling revealed by transcriptome sequencing and literature mining, TXNIP was identified as the major target gene involved in the truncated HBx-induced metabolic reprogramming. By integrated analysis of HCC patients from the TCGA database and our clinical cohort, we demonstrated that TXNIP is a potent tumor suppressor gene in HBV-induced HCC, and truncated HBx could downregulate the expression of TXNIP. Further study found that the key regulators involved in the glycolysis cascade (LDH, ENO1, ALDOA, TPI, GAPDH, PGK1) were upregulated, whereas enzymes participated in the TCA cycle (CS, PDH, MDH, IDH) were downregulated in truncated HBx-expressing cells. In addition, functional assays demonstrated that the truncated HBx decidedly promoted the glycolytic activity by increasing glucose uptake and lactate production, as well as the decreasing ATP production. Interestingly, re-introduction of TXNIP into truncated HBx-transduced cells significantly reverted the metabolic reprogramming. Furthermore, the activation of mTOR-HIF1α signaling is impeded after re-introduction of TXNIP.

HBx has been reported to transcriptionally regulate genes via transactivation through *cis*-regulatory elements [50]. Our study confirmed that the TXNIP promoter activity was repressed by truncated HBx instead of full-length HBx. To identify the precise truncated HBx-responsive motif on the TXNIP promoter, a series of truncated mutants of TXNIP promoter were constructed and subsequent site-directed mutagenesis of the potential binding motifs were performed before dual-luciferase reporter assay. NFATC2 was then identified and characterized as the transcription repressor in the promoter of TXNIP in response to the truncated HBx. Coincidently, another group previously reported that HBx can activate NF-AT in a cyclosporin A (Cs-A)-sensitive manner involving the translocation of NF-AT [35]. Since Cs-A is a canonical drug recommended by “Asian-Pacific clinical practice guidelines” on the management of hepatitis B, in the future study, we will further investigate whether Cs-A can inhibit the Ct-HBx- NFATC2-TXNIP axis to suppress HCC progression.

In conclusion, we confirmed the C-terminal truncation of X gene is a tumor-specific event during the viral integration process in the human genome and proposed a novel gene: TXNIP as a tumor suppressor gene in HBV-induced HCC by transcriptome sequencing and profiling. Most importantly, our data revealed that the truncated HBx-mediated metabolic reprogramming from mitochondrial respiration to aerobic glycolysis is in virtue of transcriptional downregulation of TXNIP via transactivation. The present study strongly demonstrated that TXNIP could serve as a potential biomarker and promising therapeutic target in HBV-induced HCC.

## Materials and methods

### HCC clinical samples

Total of 92 pairs of primary HCC clinical samples were obtained from HCC patients who underwent hepatectomy at Sun Yat-Sen University Cancer Center (Guangzhou, China). All patients enrolled were given written consent for approval of the use of clinical specimens for basic research and the study was approved by the Research Medical Ethics Committee at the hospital. All patients had completed follow-up with clinical parameters and pathologic diagnosis collected at the same time.

### Mice

In vivo mice experiments were approved by Committee of the Use of Live Animals in Teaching and Research at the University of Hong Kong. BALBc/nude mice were purchased from “Center for Comparative Medicine Research”. Details of mice model establishment can be found in Supplementary Method.

### Lentiviral production and cell transduction

Detailed procedure of plasmid construction can be found in Supplementary Method and sequence of primers are listed in Supplementary Table 1. Briefly, 293FT cells (Thermo Fisher) at 100% confluence were transfected with different HBx containing plasmids together with three packaging plasmids: Gag, Vsvg, and Rev using Lipofectamine 2000 (Invitrogen). The virus-containing supernatant were collected, and then LO2 and MIHA cells were infected and finally stably selected by 2 mM puromycin.

### Dual-luciferase reporter assay

The activity of firefly and Renilla luciferase were detected using the Dual-Luciferase Reporter Assay System (E1910, Progema). Detailed experimental procedure is provided in the Supplementary Method and primers used for PGL3 vectors construction are listed in Supplementary Table 2.

### Measurement of glucose and lactate

The indicated number of cells (For LO2 cells, 2 × 10^5^/well; for MIHA cells, 10^6^/well) were seeded in a 6-well plate with 2 mL regular DMEM medium. Forty-eight hours later, the supernatant of cells was collected for glycolysis process analysis. The level of glucose uptake and lactate secretion was determined by glucose assay kit (Nanjing Jiancheng Technology) and Lactate dehydrogenase assay kit (Nanjing Jiancheng Technology) according to the manufacture’s instruction, triplicate experiments were performed. The protein content of cells was detected to normalize the glucose and lactate level.

### Measurement of cellular ATP production

Same number of cells were seeded in triplicate in 6-well plated (For LO2 cells, 2 × 10^5^/well; for MIHA cells, 10^6^/well) and cultured for 48 h. Cells were collected and cell pellets were used for analysis. Cellular ATP production level was measured using an ATP assay kit (Nanjing Jiancheng Technology) strictly according to the manufacture’s instruction. The protein content of cells was detected to normalize the ATP level.

### RNA sequence and data analysis

A total of 1 μg RNA was extracted using RNAisoPlus (TAKARA BIO INC) and used as an original template for RNA sequencing. Pair-end 101 bp RNA sequencing was performed using the Illumina Hiseq platform at GENE DENOVO company and 6 Gb clean data was generated for each sample. Raw data were filtered for removal of adapter and low-quality reads and then mapped to the Hg38 version of the human reference genome using the BWA method, in this way the read counts of each gene for the individual sample were generated. The expression level of genes was normalized using read counts and gene length and indicated as FPKM (Fragments Per Kilobase of transcript per Million mapped reads) value. Differentially expressed genes were identified using edgeR in R studio.

### Pathway analysis

The data of RNA sequencing with the expression level of each gene in HBx-vec, HBx-120, and HBx-134 groups was uploaded to Gene Set Enrichment Analysis (GSEA) software. Both HBx-120 and HBx-134 were assigned to “Truncated HBx” group, filter parameters (normalized *P* value < 0.05, and FDR < 0.25) was preset before running the enrichment analysis. Top significant results for analysis on “KEGG pathway” and “MsigDB hallmark gene sets” were sorted based on the NES value.

### Statistical analysis

The IBM statistics SPSS 22 were used for clinicopathologic analysis, other statistical analysis was performed using Graphpad Prism 5.0 (GraphPad Software, Inc.). Procedure details are provided in Supplementary Method.

Other experimental methods including in vitro functional assay, cell cycle analysis, western blotting, IHC, and IF staining are listed in Supplementary Method.

## Supplementary information

Supplementary material
