# Correction: C-terminal truncated HBx initiates hepatocarcinogenesis by downregulating TXNIP and reprogramming glucose metabolism

**DOI:** 10.1038/s41388-021-01942-y

**Published:** 2021-07-26

**Authors:** Yu Zhang, Qian Yan, Lanqi Gong, Hang Xu, Beilei Liu, Xiaona Fang, Dandan Yu, Lei Li, Ting Wei, Ying Wang, Ching Ngar Wong, Zhaojie Lyu, Ying Tang, Pak Chung Sham, Xin-Yuan Guan

**Affiliations:** 1 Department of Clinical Oncology, The University of Hong Kong, Hong Kong, Hong Kong; 2 State Key Laboratory for Liver Research, The University of Hong Kong, Hong Kong, Hong Kong; 3 Research Center of Medical Science, Guangdong Provincial People’s Hospital, Guangdong Academy of Medical Sciences, Guangzhou, 510030 China; 4 Department of Clinical Oncology, The University of Hong Kong-Shenzhen Hospital, Shenzhen, 518053 China; 5 Department of Psychiatry, Li Ka Shing Faculty of Medicine, The University of Hong Kong, Hong Kong, Hong Kong; 6 Department of Biology, The Southern University of Science and Technology, Shenzhen, 518055 China; 7 Department of Oncology, Zhujiang Hospital, Southern Medical University, Guangzhou, 510282 China; 8 State Key Laboratory of Oncology in Southern China, Sun Yat-sen University Cancer Center, Guangzhou, 510060 China

**Keywords:** Cancer genomics, Liver cancer

Correction to: *Oncogene* 10.1038/s41388-020-01593-5, published online 15 December 2020

Unfortunately, an error occurred in Fig. 5 and in legend to Fig. 5.

**Fig. 5:**
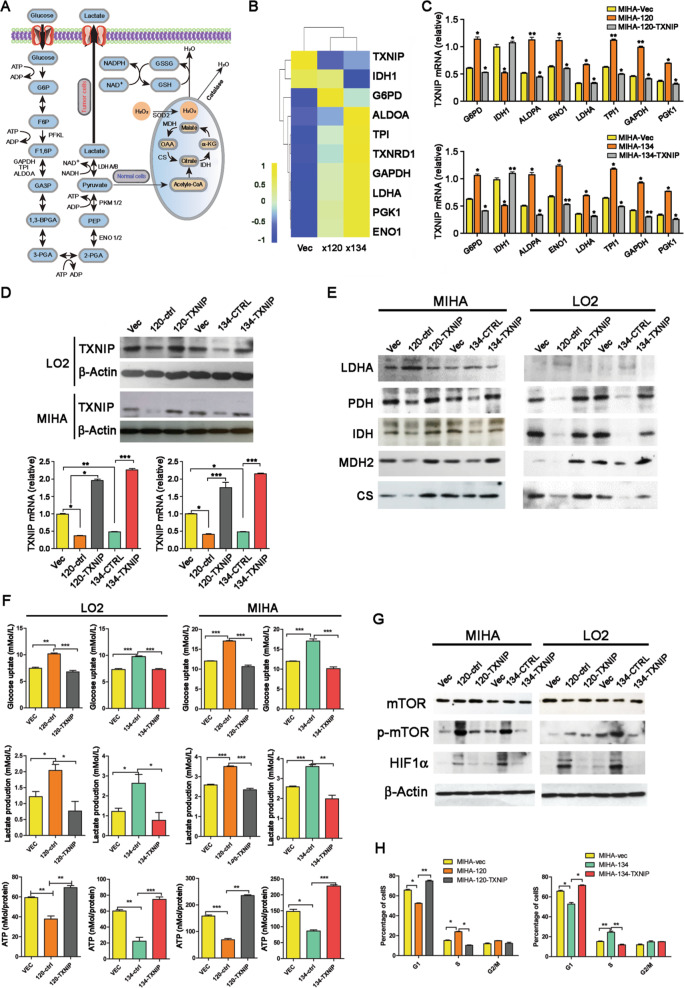
TXNIP induced glucose metabolism reprogramming from glycolysis to mitochondrial respiration. **A** Schematic representation of the biological process of glucose metabolism in normal cells and cancer cells. **B** Heatmap showing the relative expression level of several genes involved in glucose metabolism in Ct-HBx and vectors containing samples as indicated by RNA sequencing, each matrix representing the relative expression level of an individual gene; high and low expressions are indicated by yellow and blue color. **C** The expression level of the gene panel indicated above was validated by qRT-PCR in MIHA cells transduced with truncated HBx mutants compared with the vector group; also, the expression was further compared after re-introduction of TXNIP into Ct-HBx-expressing cells. **D** Re-introduction of TXNIP into Ct-HBx- (HBx-120, HBx-134) expressing cells was confirmed at the protein and genomic level by western blotting and qRT-PCR. **E** The expression level of several key enzymes and molecules that participated in glycolysis and Krebs cycle are determined by western blotting. The expression of internal reference β-actin can be referred to in (**D**). **F** Level of glucose uptake, lactate secretion, and relative ATP production activity was compared among vector, Ct-HBx as well as TXNIP overexpression samples. **G** The activation of the mTOR-HIF1α axis was detected by western blotting; β-actin was used as an internal reference. **H** Analysis of cell distribution in each stage of the cell cycle in each transfected MIHA cell.

The corrected Fig. 5 with the corrected legend is given below.

The original article has been corrected.

