# Supplementary material for: C-terminal truncated HBx initiates hepatocarcinogenesis by downregulating TXNIP and reprogramming glucose metabolism
Source: Oncogene. 2020 Dec 15;40(6):1147–61. doi: 10.1038/s41388-020-01593-5 (PMC7878188; doi:10.1038/s41388-020-01593-5)
Supplement: Supplementary file 1 — Supplementary material [file 41388_2020_1593_MOESM1_ESM.pdf]

Supplementary Methods

Cell culture

The human immortalized liver cell line LO2 and MIHA, as well as HCC cell line SNU398, SNU449, SNU182, SNU475, H2P, HepG2, H2M, SNU878, PLC-8024, were used in this study. SNU398, SNU449, SNU182, SNU878, and SNU475 cells are routinely cultured in RPMI 1640 medium (Gibco) supplemented with 10% Fetal Bovine Serum (FBS, Gibco BRL) and 100IU/mL penicillin/streptomycin (P/S), the other cells are cultured in high-glucose DMEM medium(Gibco) supplemented with 10% FBS and 1% PS. All cells were incubated in a humidified incubator at 37°C with 5% CO2 supply. Each cell line was tested for mycoplasma contamination every six months.

Cell viability and colony formation assay

1000 cells were seeded in triplicate in a 96-well plate and allowed to attach overnight, cell viability was measured by XTT kit (ROCHE) each day for 6 days. For colony formation assay, 1000 cells were seeded in a 6-well plate and allowed to grow for 10-14 days when the colony was clearly visible. Cells were washed with PBS 3 times followed by fixed with 4% paraformaldehyde for 15 minutes, then stained with 1% crystal violet (Sigma) for 15 minutes before washed carefully under tap water. The colonies were counted using light microscopy. All results in this assay are based on triple independent repeats at least.

Migration and invasion assay

For migration assay, cells were trypsinized and resuspended in the serum-free medium, diluted the cell to a certain concentration. Added 750 uL DMEM medium supplemented with 10% FBS to the lower chamber and grew cells at the appropriate density (For LO2 cells, 3×104/well; for MIHA cells, 2×104/well) in 600 uL FBS free DMEM at the upper chamber (Falcon). Cells were cultured for 24-72 hours and collected; fixed following stained according to the same protocol as colony formation assay.

For invasion assay, wells (Falcon) were pre-treated by adding 500 uL FBS-free DMEM in the upper and lower chamber and incubated for 2 hours at 37°C. Other steps were the same as migration assay, except for the cell density (For LO2 cells, 6×104/well; for MIHA cells, 4×104/well).

Mice model

Cells were pre-treated and suspended in PBS at proper concentration(For LO2 cells, 4×107/mL; for MIHA cells, 108/mL), 4-week-old BALBc/nude male mice (n=6 for treatment groups) under anesthetization were subcutaneously injected into both sides of flank with 100uL cells stably transfected with HBx or TXNIP expression plasmids (mice from the same batch were randomly allocated to each group), MIHA cells were mixed with Matrigel (50:100 cell suspension) before inoculation to increase the tumor formation rate. Mice after surgery were monitored every three days and tumor size and weight of mice were recorded by a blinded investigator. 4-6 weeks later, mice were sacrificed, and part of the fresh tumors was collected for RNA extraction and others were fixed in 4% paraformaldehyde for paraffin-embedded specimens.

Western blotting

The western blotting analysis was performed strictly followed the standard protocols of antibodies of TXNIP, TPI1, LDHA, PHD (Cell signaling technology, CST), IDH1, MDH2, CS(Santa Cruz Biotechnology), HIF1a (GeneTex), mTOR, p-mTOR (Abcam) and b-actin (Santa Cruz Biotechnology) is used as the internal reference. The whole-cell protein was extracted and standardized.

Immunohistochemical (IHC) staining

Cells attached to an 8-well chamber or paraffin-embedded tissue slides were used for IHC staining. Briefly, for tissue slides, paraffin-embedded sections (6um) were deparaffinized and rehydrated by immersing the sections into xylene, 1:1 mix of xylene and pure ethanol, each for 5 mins at room temperature, followed by 95%, 75%, 50% ethanol each for 3 mins at room

temperature. The slides then would be kept in tap water until put into 95°C retrieving buffer (10X Antigen Retrieval Buffer, diluted in ddH2O) for 45mins. For cell chambers, cells were prepared the same as IF protocol and washed in PBS followed by fixed with ice-cold 4% paraformaldehyde for 15 minutes. Then for both cell chamber and tissue slides, the endogenous peroxidase activity was blocked with 3% H2O2 for 1 hour at room temperature followed by washing in PBS 3 times; then normal goat serum was incubated for 2 hours to reduce nonspecific binding. Without washing, chambers or sections were incubated with primary antibody against TXNIP (1:100 dilution, Abcam) at 4°C overnight in a humidified box. Then slides were washed in PBS 3 times and incubated with biotinylated goat anti-rabbit IgG (Santa Cruz) for 30min at 37°C and streptavidin-peroxidase conjugated for 15min at room temperature. Finally, the 3, 5-diaminobenzidine (DAB) Substrate Kit (Dako) was used for color development followed by Mayer's hematoxylin counterstaining.

Immunofluorescence staining assay (IF)

Cells attached on the 8-well chamber were used for IF staining. Briefly, cells were seeded on chamber at 70% confluence and allowed to attach overnight. Then the chamber slides were rinsed into PBS 3 times and then fixed with ice-cold 4% paraformaldehyde for 15 minutes. Then, cells on the chamber slides were permeabilized in 0.1% Triton X-100 (Sigma-Aldrich, prepared in PBS) for 15 minutes at room temperature, followed by blocking with 5% BSA (prepared in PBS) at room temperature for 30 minutes. The slides would then be incubated with antibodies against the flag (1:100, CST) overnight at 4C. The secondary antibodies (Abcam) were used at the concentration of 1:400 in dark for 1hour at room temperature. At the last step, the slides were rinsed into PBS 3 times and stained with DAPI at the concentration of 1/1000 for 10 mins at room temperature and mounted with mounting gel for IF staining.

RNA extraction, Reverse-Transcriptase Polymerase Chain Reaction (RT-PCR) and quantitative real-time PCR (qRT-PCR)

For qRT-PCR analysis in cell lines and clinical samples, total RNA was extracted using RNAisoPlus (TAKARA BIO INC) and cDNA was synthesized by reverse transcription using PrimeScript™ RT reagent Kit (TAKARA). Then qRT-PCR was performed using a SYBR Green PCR Kit (TAKARA)and corresponding primers, Ct values were detected by ABI PRISM 7900 Sequence Detector, and data were processed using SDS 1.9.1 software. The sequence of primers are listed in Supplementary Table 1.

A set of RT-PCR primers were used to amplify different length of HBx DNA (1F: ATGGCTGCTAGGGTGTGC, 120R: TTACCAGTCCTTAAACACACAGTCT, 134R: AGATCATTAACCTAATCTCCTCCCC, 154R: TTAGGCAGAGGTGAAAAAGTTGC) using the PrimeSTAR system TAKARA BIO INC) according to the manufacturer's instruction.

Dual-Luciferase Reporter Assay

Briefly, around 2.5 kb (-2kb-500bp) of the promoter sequence of TXNIP were amplified (primer list: F: GCGTGCTAGCCCGGGCTCGAGGCCACCCAGCCTAGGCAACAGAGCAAGA; R: CAGTACCGGAATGCCAAGCTTCTTCTCTAATCAGCTTTCACCCTCC) and cloned into the pGL3-enhancer vector (Promega). 293FT cells are seeded into a white 96-well plate at the density of 4^104/ well. The luciferase reporter constructs (5:5:1 mixture of TXNIP luciferase constructs, pLenti6 truncated and full-length HBx and Renilla plasmid [Promega]) were transfected together into 293FT cells using Lipofectamine2000 (Invitrogen) according to the manufacturer’s instructions. pGL3-control vector and pGL3-enhancer vector (Promega) are used as the positive and negative controls. 48-72 hours later, add Dual-Glo® Luciferase Assay Reagent to the plate and incubate at 20–25°C for 10 minutes–2 hours before measuring firefly luminescence. Then add Dual-Glo® Stop & Glo® Reagent to the plate, incubate for the same time period before measuring the Renilla luminescence. The results were normalized to the Renilla activities. Triple wells are needed for the same experiment.

For the serial dual-luciferase reporter assay which will be applied to identify the precise binding

site on TXNIP promoter, a series of truncated mutants of TXNIP promoter(sequence of primers is listed in Supplementary Table 2) will be generated and cloned into pGL3-enhancer vector separately, and a serial of TXNIP promoter activity will be analyzed.

For site-directed mutagenesis of the potential binding motif, NFATC2, STAT3, FOXO1, and MYOG binding sites in the TXNIP promoter were mutated with a QuikChange II site-directed mutagenesis kit (Stratagene) (sequence of primers is listed in Supplementary Table. S2). The mutation of these constructs was confirmed by DNA sequencing.

Plasmid construction

Different lengths of HBV X genes with flag-tag encoding for the full-length HBx (HBx-154 primer list: F: GATTCTAGAGCTAGCGAATTCGCCACCATGGCTGCTAGGGTGTGCTG, R:ATCCTTCGCGGCCGCGGATCCTTACTTATCGTCGTCATCCTTGTAATCGGCAGAGG TGAAAAAGTTGCATG) and two Ct-HBx (HBx-120 primer list: F: GATTCTAGAGCTAGCGAATTCGCCACCATGGCTGCTAGGGTGTGCTG, R: ATCCTTCGCGGCCGCGGATCCTTACTTATCGTCGTCATCCTTGTAATCCCAGTCCTTA AACACACAGTCTTTG; HBx-134 primer list: F: GATTCTAGAGCTAGCGAATTCGCCACCATGGCTGCTAGGGTGTGCTG, R: ATCCTTCGCGGCCGCGGATCCTTACTTATCGTCGTCATCCTTGTAATCTAGTACAAAG ATCATTAACC) were cloned into pCDH-CMV-MCS-EF1α-Green vector (SBI). Linearized vectors by restriction digestion and PCR of the inserts were recombined using the ClonExpress II One Step Cloning Kit (Vazyme) according to the manufacturer's instructions.

CDS sequence of human TXNIP are amplified (primer list: F: TACTCTAGAGCTAGCGAATTCGCCACCATGGTGATGTTCAAGAAGATCAAG R: GAGGGGCGCGGCCGCGGATCCTCACTGCACATTGTTGTTGAGG) and cloned into the pLenti6/V5-TOPO vector (Invitrogen) to generate TXNIP expression plasmid.

Chromatin immunoprecipitation (CHIP) assay

Briefly, DNA and the binding protein are cross-linked using formaldehyde, and lysate will be sonicated to shear DNA to the length between 200bp to 500 bp. Then the compound will be immunoprecipitated with the antibody against the cis-regulatory element. Protein A agarose will be used to pull down the antibody-chromatin complexes. Complexes incubated with IgG are used as the negative control. Also, input will be needed without any antibody incubation. After cross-linking reversal and proteinase K treatment, DNAs will be purified by phenol-chloroform extraction and ethanol precipitation, then amplified by qRT-PCR with specific primers (sequence of primers are listed in Supplementary Table. S1) on the predicted binding site on TXNIP promoter.

Cell cycle analysis

Briefly, cells were seeded in 6-well plate at proper density (For LO2 cells, 2×105/mL; for MIHA cells, 106/mL) and allow to attach overnight, then cells were starved by cultured in FBS-free DMEM for 6-36 hours. Cells were collected at different time points (including the dead cells in the supernatant), washed in PBS, fixed with cold 70% alcohol and stained with propidium iodide (PI). Flow cytometry would be applied to determine the cell cycle distribution.

Statistical analysis

Results were presented as mean ± SEM, Student's t-test was used to compare the mean value of two groups. Pearson $\chi2$ test was applied to determine the correlation of TXNIP expression and the clinical parameters in HCC patients. Wilcoxon rank-sum test was used to compare the distribution pattern of HBV breakpoints between HCC samples and normal liver tissues. Statistical significance was defined as $P<0.05$.

For clinicopathologic analysis, the Chi-square test were applied to analyze the correlation between truncated and full-length HBx and TXNIP in clinical samples. A comparison of the mRNA level of TXNIP in paired normal and tumor tissues was by Student's t-test. Kaplan–Meier plots and log-rank tests were used for overall survival and progress-free survival analysis.

The correlation regression test was applied to evaluate the association of TXNIP expression with clinical parameters.

Supplementary Figures and Figure legends

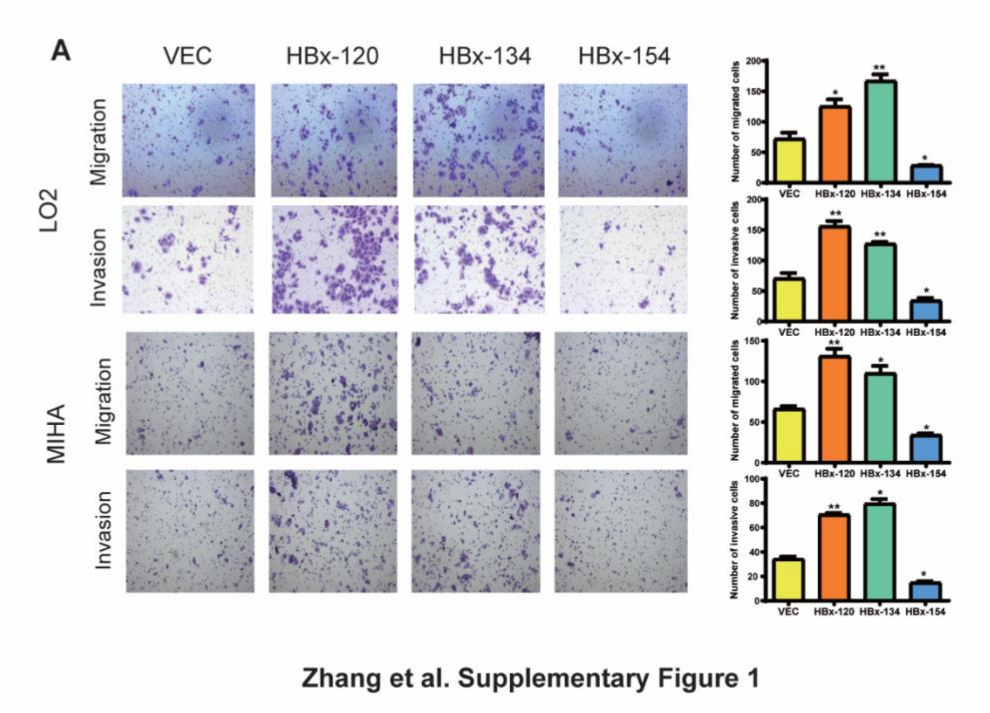

Supplementary Figure 1. Ct-HBx promote hepatocarcinogenesis

(a) Ct-HBx could significantly enhance the migrative and invasive ability of both LO2 and MIHA cells, Full-length HBx showed opposite result.

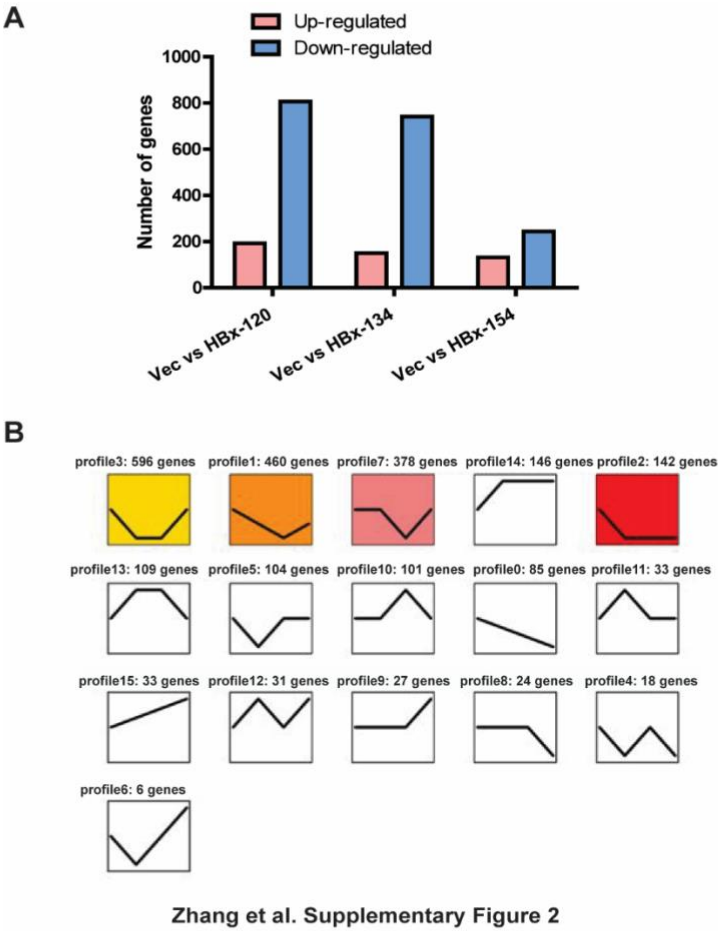

Supplementary Figure 2. Transcriptome sequencing identified TXNIP as the downstream target regulating glucose metabolism

(a) The number of differentially expressed genes between VEC vs HBx-120, VEC vs HBx-134 and VEC vs HBx-154 in LO2 cells based on the following cut-off line: fold change>1.5, FDR(adjust P value)<0.05.

(b) Tendency analysis using STEM (Short Time-series Expression Miner) revealed total 16 gene expression profiles, profiles(profile 3, 1, 7, 2) with significant P value(<0.05) which was calculated using fisher exact test were colored.

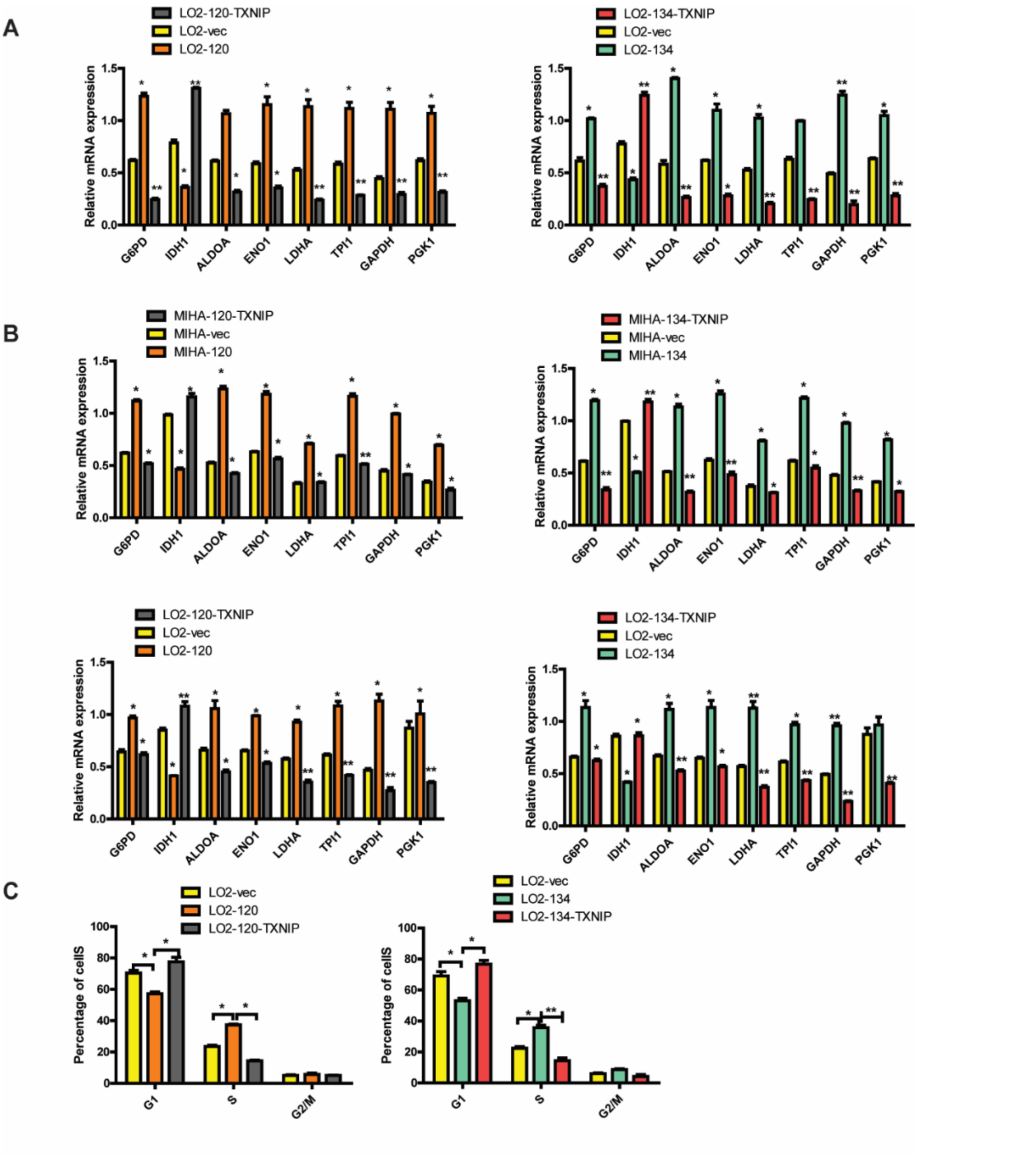

Zhang et al. Supplementary Figure 3

Supplementary Figure 3. TXNIP induced glucose metabolism reprogramming from glycolysis to mitochondrial respiration

(a) The expression level of the gene panel indicated in Fig5b was validated by qRT-PCR in LO2 cells transduced with truncated HBx mutants compared with vector group, also the expression level was compared after re-introduction of TXNIP into Ct-HBx expressing cells.

(b) The expression level of the genes was compared by qRT-PCR on RNA derived from cells-induced mice xenografts.

(c) Analysis of cell distribution in each stage of the cell cycle in each transfected LO2 cells.

Supplementary Tables

Supplementary Table 1: **Sequences of primers used in qPCR and CHIP assay**

Primer	Sequence (5'-3')
**qRT-18S-F**	AACCCGTTGAACCCCATT
**qRT-18S-R**	CCATCCAATCGGTAGTAGCG
**qRT-HBx-66F**	TCCCGCGGACGACCCGTCTCG
**qRT- HBx-100R**	CATTAACCTAATCTCCTCCCC
**qRT- HBx-154R**	GGCAGAGGTGAAAAAGTTG
**qRT-TXNIP -F**	GGTCTTTAACGACCCTGAAAAGG
**qRT-TXNIP-R**	ACACGAGTAACTTCACACACCT
**qRT-G6PD-F**	CGAGGCCGTCACCAAGAAC
**qRT-G6PD-R**	GTAGTGGTCGATGCGGTAGA
**qRT-IDH1-F**	AGAAGCATAATGTTGGCGTCA
**qRT-IDH1-R**	CGTATGGTGCCATTTGGTGATT
**qRT-ALDOA-F**	ATGCCCTACCAATATCCAGCA
**qRT-ALDOA-R**	GCTCCCAGTGGACTCATCTG
**qRT-ENO1-F**	TGGTGTCTATCGAAGATCCCTT
**qRT-ENO1-R**	CCTTGGCGATCCTCTTTGG
**qRT-LDHA-F**	ATGGCAACTCTAAAGGATCAGC
**qRT-LDHA-R**	CCAACCCCAACAACTGTAATCT
**qRT-TPI1-F**	CTCATCGGCACTCTGAACG
**qRT-TPI1-R**	GCGAAGTCGATATAGGCAGTAGG
**qRT-GAPDH-F**	ACAACTTTGGTATCGTGGAAGG
**qRT-GAPDH-R**	GCCATCACGCCACAGTTTC
**qRT-PGK1-F**	TGGACGTTAAAGGGAAGCGG
**qRT-PGK1-R**	GCTCATAAGGACTACCGACTTGG
**qRT-CHIP-F1**	AGGAGGAAGAATCCCTGCAC
**qRT-CHIP-R1**	CTGCTGGCTGTGAGATGCTT

**qRT-CHIP-F2**	AGGGTCTCTTCTGGCTTGAA
**qRT-CHIP-R2**	AGGTTTTTCCCTAAACGGTT
**qRT-CHIP-F3**	GAAGCATCCTTTTTTTCCCG
**qRT-CHIP-R3**	TTAGTGAGGTGTGTCCAGGC

Supplementary Table 2: Sequences of primers used in Dual luciferase reporter assay

Primer	Sequence (5'-3')
**TXNIP-promoter-F**	GCGTGCTAGCCCGGGCTCGAGGCCACCCAGCCTAGGCAACAGA GCAAGA
**TXNIP-promoter-R2**	CAGTACCGGAATGCCAAGCTTCCCCATTTACCTCATTACCTCTTTA
**TXNIP-promoter-R3**	CAGTACCGGAATGCCAAGCTTTCTGGTCTGAGAGCGGTGTTTT
**TXNIP-promoter-R4**	CAGTACCGGAATGCCAAGCTTGCCTAAAAAATATACGCCGCTG
**FOXO1-mutant-F**	GTCTCTTCTGGCTTGAATTTATAGTGCTCGAAGAACCGATCTTTCTTCTCTTTTCCTTCTTACGAAGTTCTAAAC
**FOXO1-mutant-R**	GTTTAGAACTTCGTAAGAAGGAAAAGAGAAGAAAGATCGGTTCTTCGAGCACTATAAATTCA AGCCAGAAGAGAG
**MYOG-mutant-F**	GCAGGGGAAAATCCATCTGAGCATCGGCCAAACGAAACCAACAAAGAATGAAG
**MYOG-mutant-R**	CTTCATTCTTTGTTGGTTTCGTTTGGCCGATGCTCAGATGGATTTTCCCCTGC
**STAT3-mutant-F**	CCTTCTTACTGTTTTCTAAACCGTTTAGTCGGCAACCTTTGAAAATAGTTTTTAAA ATTGTT
**STAT3-mutant-R**	AACAATTTTAAAAACTATTTTCAAAGGTTGCCGACTAAACGGTTTAGAAAACAGTAAGAAGG
**NFATC2-mutant-F1**	TGCACTTTTGCATACAGGTTTGGAATATTACATTTAAGAGTAAGTTCTCAGTACTCA
**NFATC2-mutant-R1**	TGAGTACTGAGAACTTACTCTTAAATGTAATATTCCAAACCTGTATGCAAAAGTGCA
**NFATC2-mutant-F2**	GCTCTGTTGACCGATCTTTCTTCTCTTGGAATTCTTACTGTTTTCTAAACCGTTTAGGG
**NFATC2-mutant-R2**	CCCTAAACGGTTTAGAAAACAGTAAGAATTCCAAGAGAAGAAAGATCGGTCAACAGAGC
**NFATC2-mutant-F3**	GAGTGAAGCATCCTTTTTTTCCCGCTTGGAATTTTCCTCCAGAAGCAGGTAGG
**NFATC2-mutant-R3**	CCTACCTGCTTCTGGAGGAAAATTCCAAGCGGGAAAAAAAGGATGCTTCACTC
